# The Microbiota-Gut-Brain Axis in Sepsis-Associated Encephalopathy

**DOI:** 10.1128/msystems.00533-22

**Published:** 2022-08-11

**Authors:** Mélanie G. Gareau

**Affiliations:** a Department of Anatomy, Physiology, and Cell Biology, School of Veterinary Medicine, University of California Davis, Davis, California, USA

**Keywords:** brain, gut, microbiota, sepsis

## Abstract

The gut microbiota is increasingly being found to contribute to the etiology and severity of multiple diseases, including within the central nervous system (CNS). This microbiota-gut-brain (MGB) axis facilitates communication between gut microbes and the brain to regulate behavior. Communication along the axis occurs via multiple routes, including the vagus nerve, gut-derived neurohormones, and immune cells, and more recently, a role for microbial metabolites has been uncovered. This commentary highlights the recent findings by H. Fang, Y. Wang, J. Deng, H. Zhang, et al. (mSystems **7**:e01399-21, 2022, https://doi.org/10.1128/msystems.01399-21) on the role of gut microbiota and bacterial metabolites in mediating sepsis-associated encephalopathy in a mouse model of cecal puncture and ligation.

## COMMENTARY

Sepsis is a serious and often life-threatening condition resulting from an inappropriate host response to bacterial or viral infection. This excessive overt peripheral inflammation in response to a pathogen or pathogen-associated molecular patterns (PAMPs) can lead to neurological impairments in a subset of patients, including cognitive deficits and anxiety-like behavior. These behavioral impairments can occur even in the absence of infection within the central nervous system (CNS). Although these neurological complications are associated with poor prognosis in sepsis patients (1), including sepsis-induced encephalopathy (SAE), it is uncertain that these are causative or simply reflective of severe illness. Despite this outstanding question of causality or correlation, identification of pathways that trigger these neurological complications are important in improving overall patient care. Given the strong association between the microbiota, gut, and brain, coupled with the known detrimental impact of pathogen infection on composition of the gut microbiome, it is conceivable that altered host-intestinal microbe interactions lead to SAE.

The microbiota-gut-brain (MGB) axis is a bidirectional communication pathway that is established beginning in early life and is crucial for regulating overall health and homeostasis (2). Studies using germfree (GF) mice have identified that the gut microbiota is important for maintaining behavior and cognitive function, in part by regulation of myelination (3), neurogenesis (4), and microglia (5) within the CNS. While precise mechanisms of communication within the MGB axis remain to be fully elucidated, bacterial metabolites, gut-derived neurohormones, the vagus nerve, and immune cells all have strong evidence for playing a role in maintaining this communication (2). Therefore, given its important role in maintaining gut-brain signaling, the gut microbiota represents a possible mechanism via which a systemic infection may lead to neurological deficiencies within the CNS.

The composition of the gut microbiota is dynamic in early development, stabilizing in children by approximately age 5 (6). While the patterns of colonization can be negatively impacted in multiple disease states, the functional changes regulated by altered gene expression within the span of the entire community may be more important in maintaining appropriate host-microbe interactions. There are many mechanisms of interkingdom communication that have been proposed to allow bacteria to communicate within the mammalian host. For example, bacterial metabolites are increasingly recognized as a means by which bacterial-host interactions are maintained. There are several proposed mechanisms through which these metabolites are thought to be able to induce responses in mammalian cells, including via the expression of cognate receptors to which these bacterially derived metabolites can bind and either activate or inhibit downstream signaling pathways. Perhaps unsurprisingly, there are a multitude of bacterial metabolites that have been identified to change host physiology, including short-chain fatty acids (SCFA). These compounds are produced by multiple beneficial bacterial strains and function to not only maintain colonocyte health but also immune function both peripherally and in the brain, including regulation of microglia function as demonstrated in studies using GF mice (5). In addition to the well-characterized SCFA, indole-3-propionic acid (IPA) produced by Clostridium sporogenes has also been identified as a neuroprotective functional metabolite (7). Similar to SCFA, IPA crosses the blood-brain-barrier and can beneficially impact astrocyte and microglia function in the brain.

In the article by Fang et al. (8), induction of sepsis via cecal ligation and puncture (CLP) was used to study a potential role of the MGB axis in controlling disease progression. In their model, a subset of animals developed significant neurological deficits, as determined by a composite score of pinna reflex, corneal reflex, righting reflex, tail flexion reflex, and escape response, with lower scores (<6) associated with poor prognosis, including mortality. Mice that were susceptible to SAE (sepsis-induced encephalopathy susceptible [SES] mice) had an intestinal microbiota that was enriched for the Gram-negative *Enterobacteriaceae* family, which is known to promote inflammation and lead to sepsis (8). Demonstrating a role of the microbiota in dictating these poor neurological outcomes during sepsis, fecal microbiota transplantation (FMT) from mice resistant to SAE (sepsis-induced encephalopathy resistant [SER] mice) was protective in recipient SES mice, validating a role for gut microbes in SAE. This role of the microbiota was further substantiated by transfer of the SES phenotype induced by CLP to normally resistant (SER) mice via FMT (8). Mechanistically, the bacterial metabolite IPA was found to be enriched in the feces from SER mice, suggesting that IPA may protect against CNS deficits seen in SAE. Indeed, administration of IPA could protect mice against CLP-induced SAE and death, which was thought to be mediated in part by inhibiting the activation of the NLRP3 (NOD-, LRR- and pyrin domain-containing protein 3) inflammasome in microglia. Additionally, in *in vitro* studies where primary microglia were isolated from the cortex of neonatal mice pretreated with IPA and exposed to the bacterial endotoxin lipopolysaccharide (LPS), IPA reduced proinflammatory cytokine production and apoptosis (8). Finally, since the aryl hydrocarbon (AhR) can negatively regulate activity of the NLRP3 inflammasome, the authors discovered that pretreatment with an AhR antagonist could inhibit the beneficial impacts of IPA on microglia *in vitro*. The authors thus hypothesized that treatment with IPA inhibits activation of NLRP3, which could be reversed by blocking AhR signaling using an antagonist. Overall, this study highlights important features delineating that variability of sepsis-induced gut dysbiosis may mediate the differential susceptibility to SAE following induction of sepsis and reveals possible mechanisms, including decreased levels of microbially derived IPA, to explain why these neurological impairments are not uniform across all animals. These findings may uncover future novel clinical parameters in which one can easily identify which patients are more at risk for SAE than others.

While this study by Fang et al. (8) is novel and exciting, providing mechanistic insights into the role of the gut microbiome in regulating SAE, limitations exist. First, the effect of IPA on ameliorating survival and neurological scores in mice is partial, suggesting that other mechanisms are also contributing to the impacts of SAE. In addition, many SAE-associated behavioral impairments were not alleviated by IPA treatment, suggesting that neuroinflammation was not the primary cause of these particular SAE-induced complications. Whether these are maintained by additional bacterial metabolites or other unrelated pathways remains unclear. Lastly, the specific mechanism by which IPA blocks NLRP3 activity was not fully delineated in the current study and remains of interest to elucidate. Future studies will hopefully fully define the signaling pathways regulating the risk for development of SAE in critical patients, including the role of the MGB axis, and provide new tools to improve patient outcomes.
